# Non-invasive brain stimulation for treating catatonia: a systematic review

**DOI:** 10.3389/fpsyt.2023.1135583

**Published:** 2023-05-16

**Authors:** Hongqi Xiao, Yajing Meng, Shiyu Liu, Yuan Cao, Huan Sun, Gaoju Deng, Mei Wang, Yaozong Zheng, Changjian Qiu

**Affiliations:** ^1^Mental Health Center, West China Hospital of Sichuan University, Chengdu, China; ^2^Sichuan Clinical Medical Research Center for Mental Disorders, Chengdu, China; ^3^Department of Nuclear Medicine, West China Hospital of Sichuan University, Chengdu, Sichuan, China; ^4^Department of Radiology, Huaxi MR Research Center (HMRRC), West China Hospital of Sichuan University, Chengdu, China

**Keywords:** non-invasive brain stimulation, catatonia, electroconvulsive therapy, repetitive transcranial magnetic stimulation, transcranial direct current stimulation

## Abstract

**Background:**

Non-invasive brain stimulation (NIBS) techniques offer new therapeutic options for modifying pathological neuroplasticity and have been proven to be beneficial in the treatment of neuropsychiatric disorders.

**Objective:**

This study aimed to investigate the role of NIBS in treating catatonia.

**Materials and methods:**

We conducted a systematic search to identify meta-analyses or systematic reviews on electroconvulsive therapy (ECT) and studies on the effects of repetitive transcranial magnetic stimulation (rTMS) and transcranial direct current stimulation (tDCS) on patients with catatonia from the PubMed, Web of Science, Embase, China National Knowledge Internet, Wanfang, and China Science and Technology Journal databases from inception until 31 July 2022. The methodological quality of the included studies was assessed with the AMSTAR2 or Joanna Briggs Institute Critical Appraisal tools. Paired *t*-tests and Wilcoxon signed-rank tests were used to compare changes in catatonia symptom scores after rTMS or tDCS.

**Results:**

A total of 13 systematic reviews and one meta-analysis on ECT, two systematic reviews and 12 case reports on rTMS, and seven studies of 14 cases applying tDCS were identified. Systematic reviews of ECT consistently described improvement in catatonia symptoms across catatonia types and patient age groups. After treatment with rTMS (*t* = 4.489, *p* = 0.006) and tDCS (*z* = −3.065, *p* = 0.002), patients exhibited significant improvement.

**Conclusion:**

ECT, rTMS, and tDCS were effective in treating catatonia. Early intervention with NIBS techniques may help improve catatonia symptoms in patients with schizophrenia. It may be advantageous to use rTMS or tDCS to maintain this improvement. NIBS techniques may thus represent a promising treatment for catatonia, but additional high-quality randomized controlled trials are needed.

## 1. Introduction

Catatonia is a severe volitional and psychomotor syndrome characterized by symptoms of stupor, mutism, immobility, rigidity, negativism, stereotypy, posturing, and mannerism. It can occur in patients with schizophrenia, mood disorders, and somatic conditions. The prevalence of catatonia in psychiatric inpatients is estimated to be between 10 and 16% (1, 2). Catatonia can lead to dangerous complications, including severe nutritional deficiencies, aspiration pneumonia, dehydration, urinary tract infections, electrolyte disturbances, and venous thromboembolism (3, 4). Without proper treatment, catatonia can result in high mortality rates (5, 6). Therefore, early diagnosis and treatment are crucial (7), and physicians need to be knowledgeable about the clinical characteristics of catatonia and the available treatment options.

To emphasize the importance of diagnosing catatonia, catatonia was categorized under a separate heading in the “Schizophrenia Spectrum and Other Psychotic Disorders” chapter in the Diagnostic and Statistical Manual of Mental Disorders, 5th edition (DSM-5). The new diagnostic criteria for catatonia suggest that catatonia is a separate neuropsychiatric syndrome. The proper diagnosis and treatment of catatonia are crucial and are receiving increasing attention from clinicians.

Although the etiology and pathophysiology of catatonia are not clear, previous studies have described brain circuit dysfunction in patients with catatonia. Specifically, several studies have reported that catatonia is associated with alterations in cerebral motor circuits (8–10). A recent review highlighted that premotor hyperactivity is an important pathophysiological feature of catatonia (11). In addition, reduced neural activity in the frontal and parietal cortices has been identified in patients with catatonia (8, 12). These findings suggest that therapeutic approaches to modulate neurological dysfunction may be beneficial in the treatment of catatonia.

Non-invasive brain stimulation (NIBS) techniques such as electroconvulsive therapy (ECT); repetitive transcranial magnetic stimulation (rTMS), including theta burst stimulation (TBS); and transcranial direct current stimulation (tDCS) represent therapeutic options for the modification of pathological neuroplasticity. Previous studies have shown that NIBS can induce or modify plasticity in the human central nervous system, with various functional effects on cognitive, emotional, and motor processes (13–17). In addition, the therapeutic effects of NIBS on neuropsychiatric disorders have been confirmed by numerous studies (18–23). Thus, the modulation of neuronal activity by the NIBS technique supports its application in the treatment of catatonia. Many studies have explored the efficacy of ECT treatment for patients with catatonia (24–27), but the results are varied and heterogeneous. Systematic reviews can present a more comprehensive picture of the current research progress on ECT for the treatment of catatonia and provide some clinical ideas and references. Therefore, we conducted an overview of systematic reviews and meta-analyses regarding the efficacy of ECT for treating catatonia. Considering the limited number of studies using rTMS and tDCS in the treatment of patients with catatonia and that the protocols and efficacy of rTMS and tDCS in the treatment of catatonia remain unclear, a systematic review of original studies of rTMS and tDCS was conducted to evaluate the protocols and efficacy of rTMS and tDCS in the treatment of catatonia. Overall, our study aimed to determine the role of NIBS techniques in the management of catatonia.

## 2. Materials and methods

### 2.1. Search strategy

This systematic review was conducted according to the Preferred Reporting Items for Systematic Reviews and Meta-Analyses (PRISMA) statement (http://www.prismastatement.org/) (28).

A comprehensive literature search was performed independently by two reviewers (HX, GD) on 31 July 2022. Searches were performed in six different electronic databases: PubMed, Web of Science, Embase, China National Knowledge Internet, Wanfang, and China Science and Technology Journal. The search strategy included the use of Medical Subject Headings (MeSH) terms and keywords, and the detailed search strategies are shown in the Supplementary material. Moreover, the references of the identified records were inspected to identify additional relevant studies.

### 2.2. Inclusion and exclusion criteria

Studies included in this review were chosen based on the Population, Intervention, Comparison, Outcomes, and Study (PICOS) elements. Studies published in English and Chinese were considered. For rTMS and tDCS studies, the inclusion and exclusion criteria were as follows:

- *Study participants:* For patients diagnosed with catatonia, there were no restrictions on diagnostic composition or participant age.
- *Study intervention*: Treatment with rTMS (including iTBS) or tDCS must be used as the intervention measure. Catatonia could be treated with rTMS or tDCS alone or combined with medicine or other treatments, regardless of the frequency or duration of the treatment. Literature that did not report treatment parameters such as stimulation intensity or the number of sessions was excluded.
- *Study comparison*: Studies with or without control interventions were included.
- *Study outcome measures:* The primary outcomes were the severity of catatonia measured by clinical judgment or using one of the following catatonia or clinical improvement rating scales: the Bush-Francis Catatonia Rating Scale (BFCRS), Kanner Catatonia Rating Scale (KCRS), Clinical Global Impression scale (CGI), Modified Rogers Scale (MRS), or Brief Psychiatric Rating Scale (BPRS). Secondary outcomes were the percentage values for catatonia symptom reduction and adverse events.
- *Study design:* Published case reports, case series, cohort studies, cross-sectional studies, case-control studies, randomized clinical studies, systematic reviews, or meta-analyses were included.

For ECT articles, the inclusion and exclusion criteria were as follows:

- *Study participants:* For patients diagnosed with catatonia, there were no restrictions on diagnostic composition or participant age.
- *Study intervention:* Treatment with ECT must be used as the intervention measure. Catatonia could be treated with ECT alone or combined with medicine or other treatments, regardless of the frequency or duration of the treatment.
- *Study comparison:* Studies with or without control interventions were included.
- *Study outcome measures:* The primary outcomes were the response rate of catatonia symptoms assessed by the BFCRS, KCRS, CRI, MRS, or BPRS, or clinical judgment of catatonia symptom improvement. The secondary outcomes were adverse events.
- *Study design:* Published systematic reviews or meta-analyses were included. Review comments, overviews of systematic reviews, editorials, and guidelines were excluded. Reviews involving the exploration of the treatment response to ECT in patients with catatonia were included.

### 2.3. Study selection

The organization and exclusion of duplicated articles were performed by EndNote X9. After the removal of duplicate articles, two phases were established to select the studies. In the first phase, two authors (MW and YZ) screened the abstracts of identified articles to remove irrelevant articles; any discrepancies were resolved by a third author (YM). The second phase involved two authors (HX and SL) who reviewed the full texts of the publications. Disagreements were resolved by discussion until a consensus was reached among three reviewers (HX, SL, and CQ).

### 2.4. Data extraction and analysis

Data extraction was led by two independent reviewers (HX and YC), and disagreements were resolved through consensus with the senior author (HS). For studies on ECT treatment for catatonia, the extracted information included the study title, patient population (a subtype of catatonia), the studies included (i.e., for meta-analyses), ECT parameters, evidence of ECT treatment of catatonia, evidence quality, conclusions, and appraisals. Since only one relevant meta-analysis was identified for inclusion, we only summarized the results and did not perform the data analysis.

For studies of tDCS and rTMS, the following information was extracted: study characteristics (authors and publication year), patient characteristics (diagnosis, age, and sex), BFCRS scores, treatment parameters (e.g., targeted brain region, intensity, and the number of sessions), symptom improvement, adverse effects, and reasons for receiving NIBS. The percentage values for symptom reduction were calculated by subtracting the post-treatment BFCRS score from the pre-treatment BFCRS score and then dividing it by the pre-treatment BFCRS score.

Except for systematic reviews, all eligible studies of tDCS and rTMS were case studies; therefore, to reflect the overall treatment efficacy of tDCS or rTMS, we used paired *t*-tests and Wilcoxon signed-rank tests to compare the BFCRS scores before and after treatment, referring to a previous study (29). If one study reported a range of BFCRS scores, the mean score was used for further analyses. To compare the treatment efficacy between tDCS and rTMS, we performed independent-sample *t*-tests to compare the treatment-induced changes in the BFCRS scores between groups.

**Figure 1:**
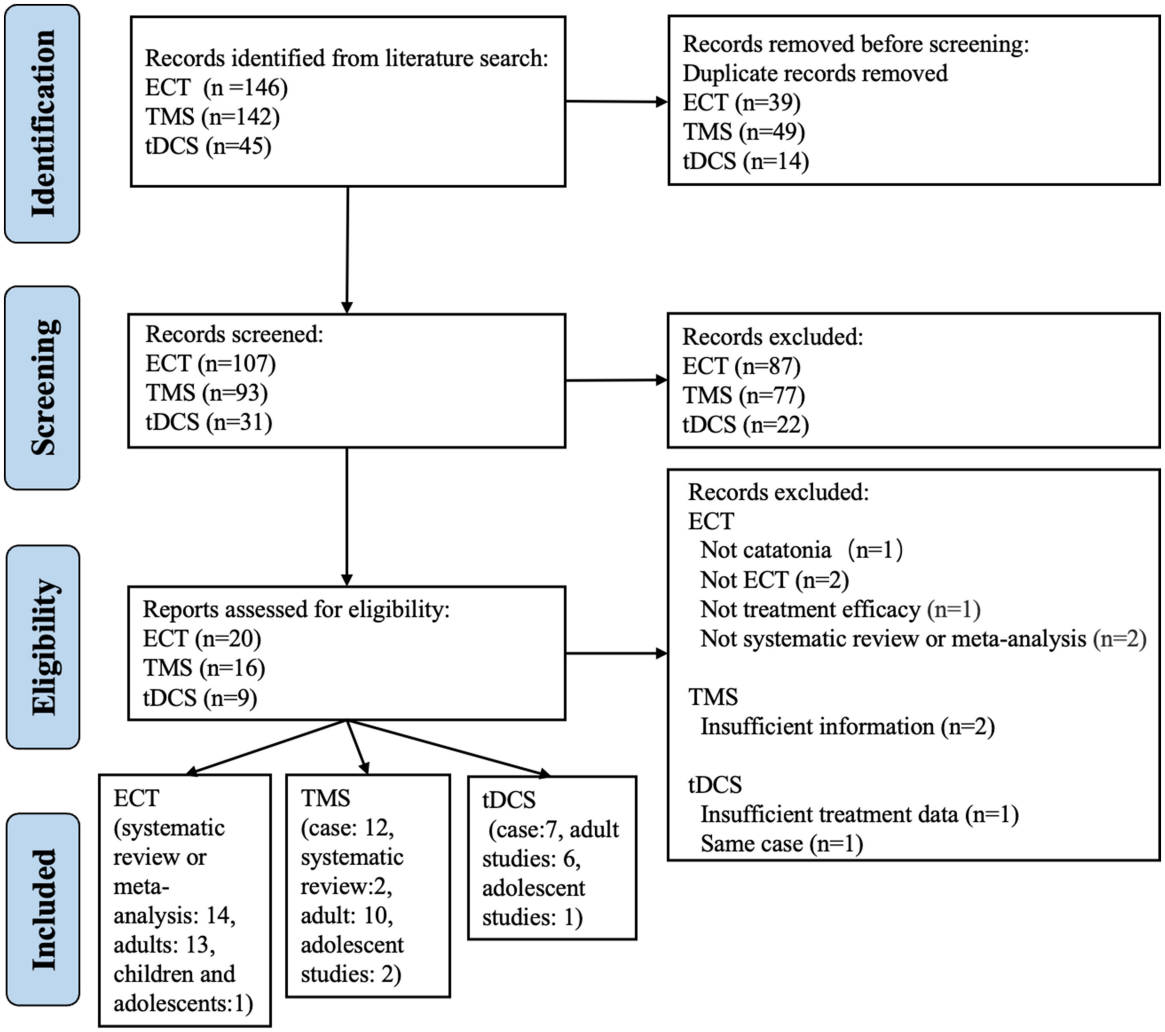
PRISMA flow diagram.

The initial literature search for studies of catatonia and ECT identified 146 records, of which 39 were duplicates. After removing duplicate records, we screened the titles and abstracts of the remaining 107 articles. A total of 87 irrelevant articles were excluded. Subsequently, 20 full-text articles were assessed for eligibility. Ultimately, 14 studies were included in this review (30–43) (Figure 1).

In the initial literature search for studies of catatonia and TMS, 142 records were identified. After removing duplicate records, 93 titles and abstracts were screened; of these, 77 were excluded due to irrelevance. The full texts of the remaining 16 articles were assessed for eligibility, and two studies were excluded due to insufficient information (44, 45). Ultimately, 14 articles were included in this review (29, 46–58). Among these studies, two were systematic reviews (29, 58). The first of these systematic reviews, by Beach et al. (58), investigated alternative treatment strategies for catatonia and included four articles on rTMS and one article on tDCS. Due to the limited number of studies included, we did not conduct a quantitative analysis in this systematic review. The other systematic review (29) included nine case studies of rTMS and four case studies of tDCS and included an in-depth analysis; however, this systematic review did not investigate the efficacy of ECT treatment for catatonia. The remaining 12 studies included in our analysis were case reports, of which two were conference abstracts (48, 50); all 12 were included in the quantitative analysis, as shown in Figure 1.

The literature search for studies of catatonia and tDCS returned 45 records, of which 14 were duplicates; the remaining 30 reports were screened. Of these, 22 studies were excluded after screening the titles and abstracts, the full texts of nine reports were further assessed for eligibility, and one study was excluded due to insufficient treatment data (59) (i.e., the tDCS stimulation protocol was not described). Of the remaining eight reports, Weidinger (60) and Keeser et al. (61) described the same patient and reported results after approximately 66 sessions and 330 sessions of tDCS, respectively; therefore, we included only the latter. Hence, a total of seven studies were included in our systematic review (61–67) (see Figure 1).

### 2.5. Methodological quality/risk of bias assessment

Two reviewers (HX and YM) assessed the methodological quality of the included systematic review and meta-analyses using A Measurement Tool to Assess systematic Reviews (AMSTAR-2) (68) and the risk of bias of the included case studies using the Joanna Briggs Institute Critical Appraisal Tools for case reports and case series (69). Disagreements were resolved by a third reviewer (QC). The AMSTAR-2 is a 16-item questionnaire, and the overall rating is based on weaknesses in critical domains (Items: 2, 4, 7, 9, 11, 13, and 15) as follows: “high,” which indicates no or one non-critical weakness; “moderate,” which indicates more than one non-critical weakness but no critical flaws; “low,” which indicates one critical flaw with or without non-critical weaknesses; and “critically low,” which indicates more than one critical flaw with or without non-critical weaknesses. Case studies assessed by Joanna Briggs Institute Critical Appraisal tools were evaluated based on the following criteria: Studies with more than 70% of the items scored as “yes” were considered to have a low risk of bias; studies with 50%−69% of the items scored as “yes” were considered to have a medium risk of bias; and studies with < 49% of the items scored as “yes” were considered to have a high risk of bias. As recommended by the JBI reviewers' manual, all decisions regarding the scoring system and cutoff points were approved by all reviewers before the start of the critical appraisal process.

## 3. Results

### 3.1. ECT for catatonia

We provided information regarding ECT treatment for catatonia and summarized the findings of the included meta-analyses and systematic reviews (Table 1). In total, 13 of the identified articles were systematic reviews (30–35, 37–43), and only one study (36) included a meta-analysis of the efficacy of ECT treatment in patients with catatonia. Of these, three studies summarized the effects of ECT on patients with unrestricted catatonia (36, 38, 40) (i.e., all types of catatonia), and 11 studies described the benefits of ECT for catatonia patients with the following different etiologies: catatonic schizophrenia [two reviews (37, 41), including 35 studies], autistic spectrum disorder [ASD; two reviews (32, 42)], malignant catatonia (31), benzodiazepine withdrawal (39), anti-N-methyl-d-aspartate (NMDA) receptor encephalitis (43), coronavirus disease 2019 (30) (COVID-19), and obsessive–compulsive disorder (OCD) (35). Additionally, one review focused on ECT for catatonia in children and adolescents (33), and another focused on catatonia in older adults (34).

**Table 1:** Evidence from systematic reviews for ECT treatment of catatonia.

**Study**	**Patient population**	**Studies included**	**ECT parameters**	**Evidence for ECT treatment of catatonia**	**Evidence quality**	**Conclusion**	**Appraisal**
Leroy et al. (36)	Catatonia	Total: 28 studies RCTs: 3 Cases: 25 (one case series nested in an RCT, 12 prospective cases and 12 retrospective cases)	**Sessions:** 3–35 (average: 9) **Sites:** BL: 5, BTL: 5, BFT: 2, BF: 1, BF vs. BTL: 1, BL or BL+UL: 1, BFT or UL: 1, left anterior right temporal/BTL: 1, not described: 9 **Frequency:** 2–5 sessions/week, 11 studies performed 3 sessions/week	**Response rates:** 42%−100% **Side effects** (28.6%): mental confusion, headache, memory loss, or adverse effects associated with anesthesia ***Loss of memory was the most reported adverse effect*** Ten studies of 211 participants included in quantitative analysis showed an SMD of −3.14 [95% CI (−3.95, −2.34)] and demonstrated improvement in catatonia symptoms after ECT	Bias varied from low to high and heterogeneity was high (*I*^2^ = 76.6%, Q test: *p* < 0.001)	Improvement in catatonia symptoms after ECT were consistently described; however, there were **insufficient high-quality RCTs** to demonstrate ECT efficacy, tolerance or protocols in catatonia	**This review was the first and only meta-analysis of ECT treatment for catatonia**; however, the RCTs included were scarce and of low quality
Luchini et al. (38)	Catatonia	Total: 8 studies including at least 10 patients Retrospective cases: 7 Observational cases: 1	**Sessions:** 12–20 **Sites:** BTL: 4, BF: 1, BL: 1, BFT or UL: 1, Not described: 2 **Frequency:** 3 sessions/week	**Response rates:** 80%−100% ***ECT produced superior results in catatonia patients than other psychiatric therapies ECT was an effective treatment for all types of catatonia, even after pharmacotherapy was unsuccessful***	Not performed	**ECT should be considered as first-line treatment** for patients with MC, NMS, and delirious mania or severe excited catatonia as well as patients refractory/partially responsive to benzodiazepines **Early intervention with ECT is encouraged** to prevent unnecessary deterioration of a patient's medical condition	The efficacy, administration technique, safety, combined treatment, and maintenance treatment for catatonia were described in detail. However, this review did not include a quantitative analysis
Pelzer et al. (40)	Catatonia	Total: 11 studies Prospective cohort studies: 2 Retrospective cohort studies: 5 RCT: 1 Prospective, observational study: 1 Case series: 1	**Sessions:** 2–13 **Sites:** BL: 4, UL: 2, BTL: 1, not described: 4 **Frequency:** 3 sessions/week in 6 studies, not described in 5 studies	**Response rates:** 59%−100% **Side effects:** cognitive/memory impairment, headache during treatment *ECT was initiated **not only** after ineffective pharmacotherapy **but also as a primary therapy***	Not performed	**ECT was a very effective treatment for catatonia**, including after the failure of benzodiazepines (lorazepam) **ECT may be a good alternative for pharmacotherapy in life-threatening catatonia cases**	This review not only described the treatment efficacy of ECT but also the efficacy of drugs. However, this review did not include a quantitative analysis
Liu et al. (37)	Catatonic schizophrenia	Prospective case series: 4	**Sessions**: 12: 2, not described: 2 **Sites:** BL **Frequency:** not described	ECT appeared well-tolerated and successful for treating catatonic schizophrenia ***Acute ECT was efficacious before and after relapse***	Not performed	**Bilateral ECT seemed effective both as acute and maintenance treatment in older patients** with schizophrenia	This review summarized **the efficacy of ECT for late-life catatonic schizophrenia and the use of ECT when patients relapsed**. However, the number of studies included in this review was low
Pompili et al. (41)	Catatonic schizophrenia	Total: 3 studies Cohort study: 1 Observational study: 1 Prospective study: 1	**Sessions:** mean of 8.4, not described: 5 **Sites:** not described **Frequency:** not described	**Response rate:** 100% ***Patients with catatonic schizophrenia responded faster to ECT than patients with noncatatonic schizophrenia*** The most common symptom of schizophrenia was catatonia The most common reason of ***the use for ECT was to augment pharmacotherapy***	The total quality scores of the three studies were 3, 4, and 5, indicating low quality	This research concluded that catatonic patients responded significantly better to ECT than patients with any other subtype of schizophrenia. **The use of ECT was recommended for schizophrenia patients with catatonia**	This review revealed that catatonic schizophrenia patients responded better to ECT than those with other subtypes of schizophrenia. However, it included only three studies of catatonia and did not perform a quantitative analysis
Cronemeyer et al. (31)	Malignant catatonia (MC)	BZD and ECT: 45 case reports; ECT: 23 case reports (BZD: 24 case reports; Antipsychotics: 8 case reports; Supportive therapy: 11 case reports)	**Sessions:** not described **Sites:** not described **Frequency:** not described	***No deaths and the highest rate of cases with full alleviation of symptoms (76.7%) were observed in patients treated with both BZD and ECT*** compared to those that received antipsychotics and supportive therapy, respectively Rates of full alleviation of symptoms due to ECT did not vary significantly among therapy groups, nor did the rates of partial remission	Not performed	**The most favorable outcome in MC cases was due to administration of both benzodiazepines and ECT**	This systematic review was **the first to focus on the treatment efficacy of ECT in patients with MC**. It compared the effectiveness of different treatment approaches for MC in terms of outcome and severity; however, all studies included in this review were case reports
DeJong et al. (32)	Catatonia in ASD patients	11 case studies	**Sessions:** 7–29 **Sites:** BL: 9, BL or UL: 2 **Frequency:** 3 sessions/week to one session every 2–3 weeks	Almost all patients exhibited marked or dramatic improvement with ECT Several patients reported rapid recurrence of symptoms when ECT was discontinued or suspended. Follow-up periods were not always clearly reported but appeared to range from 4 weeks to at least 14 months **Side effects:** [1] Most studies did not report adverse effects of ECT. One patient reported “mild delirium,” one reported increased symptoms, and another reported prolonged seizure	Data quality was rated using a checklist developed for the purpose of this review. Scores ranged from 4 to 9, indicating low quality	There may have been an initial response to ECT, resulting in partial alleviation of catatonia symptoms, but this effect appeared to be temporary	This review summarized the treatment efficacy, side effects and maintenance treatment of ECT for ASD patients with catatonia symptoms in detail; however, all studies were case reports with poor quality of evidence
Vaquerizo-Serrano et al. (42)	Catatonia in ASD patients	2 retrospective longitudinal studies	**Sessions:** not described **Sites:** not described **Frequency:** not described	The use of short-term and maintenance ECT might be beneficial ***Maintenance ECT was necessary for sustained symptom alleviation***	Evidence quality was evaluated using a modified version of the Newcastle–Ottawa Scale. The score of both studies was 4	ECT improved catatonia symptoms in ASD patients	**ECT was less frequently reported as a treatment for ASD patients with catatonia**
Jaimes-Albornoz et al. (34)	Catatonia in OCD patients	10 case studies	**Sessions:** 8–21 **Sites:** not described **Frequency:** not described	Four of 10 cases achieved complete alleviation of catatonia Response was obtained only after optimization of the OCD treatment with medication, ECT, and psychotherapy (alone or combined), and over various weeks	Not performed	This review highlights **the importance of actively treating the underlying etiology of catatonia**. The treatment of catatonia etiology led to alleviation of symptoms more frequently than treatment of specific symptoms alone	This review focused on ECT treatment for catatonia in OCD patients and indicated the importance of etiology treatment. However, only case studies were reported in this review
Austgen et al. (30)	Catatonia in COVID-19 patients	2 cases	**Sessions:**9–10 **Sites:** not described **Frequency:** not described	A case in which ECT was used to ***achieve alleviation of symptoms*** in a patient who developed catatonia associated with COVID-19 was reported. Another case diagnosed with COVID-19 and catatonia ***achieved full recover**y* after ECT treatment	Not performed	ECT was safe and effective for new neuropsychiatric symptoms associated with COVID-19	This was the first **review to report the treatment efficacy and safety of ECT for COVID-19-associated catatonia**. However, there were only 2 cases, and the evidence quality was poor
Oldham and Desan (39)	Withdrawal catatonia	1 case study	**Sessions:** not described **Sites:** not described **Frequency:** not described	One case of ***benzodiazepine-withdrawal catatonia*** was reported; ECT had good treatment efficacy	Not performed	Withdrawal catatonia was responsive to ECT	This review only reported 1 case (a patient with benzodiazepine-withdrawal catatonia) who was administered ECT
Warren et al. (43)	Catatonia in anti-NMDA receptor encephalitis patients	25 case studies of 26 cases	**Sessions:** 1–33 **Sites:** BL: 1, BTL: 2, not described: 22 **Frequency:** not described	17 cases received ECT treatment and had reported treatment outcomes. Among the 17 cases, 5 (29.4%) had full alleviation of catatonia symptoms, 8 (47.1%) had improvement in catatonia symptoms after ECT, and 4 (23.5%) had no improvement of catatonia symptoms	Not performed	ECT appeared to be an effective and safe adjuvant treatment in anti-NMDA receptor encephalitis patients, particularly for catatonia patients	This review reported the treatment efficacy of ECT for anti-NMDA receptor encephalitis in patients with catatonia symptoms; however, all studies were case reports
Døssing et al. (33)	Catatonia in children and adolescents	Total: 23 studies Retrospective studies: 2 Case series: 6 Case studies: 15	**Sessions:** not described **Sites:** not described **Frequency:** not described	***ECT had high treatment response rates for catatonia in children and adolescents***	The quality of evidence according to the Grading of Recommendations, Assessment, Development and Evaluations was rated low to very low for studies included in this review	ECT should be considered for catatonia in children and adolescents	This review comprehensively summarized studies of catatonia in children and adolescents treated with ECT; however, there were no RCTs, and a quantitative analysis was not performed
Jaimes-Albornoz et al. (35)	Catatonia in older adults	Unspecified number of included studies on catatonia in older adults treated with ECT	**Sessions:** 2–25 **Sites:** BFT: most cases, UL (ambiguous number of studies), BFT switch to BTP: 1 **Frequency:** 2 or 3 times a week	***Catatonia treatment with ECT is safe and effective in older patients*** Three older patients with catatonia associated with general medical conditions had only a partial response to ECT after receiving 7–8 sessions Better response rates for older patients with catatonia treated with ECT were reported for mood disorders than nonaffective psychosis	Not performed	ECT was safe and effective for treating catatonia in elderly individuals. Medical risks should be evaluated on an individual basis	This review focused on catatonia symptoms in older adults and did not report the number of studies of older catatonia patients treated with ECT

ECT, electrotherapy; RCT, randomized controlled trial; SMD, standard mean difference; MC, malignant catatonia; BZD, benzodiazepine; ASD, autism spectrum disorder; OCD; obsessive-compulsive disorder; COVID-19, coronavirus disease 2019; NMDA, anti-N-methyl-d-aspartate; UL, unilateral; BL, bilateral; BTP, bilateral temporoparietal; BFT, bilateral frontotemporal; BTL, bilateral temporal. The bold format emphasizes important results.

Among the three reviews (36, 38, 40) that summarized the effects of ECT on unrestricted catatonia, the systematic review conducted by Leroy et al. (36) included the largest number of studies [28 studies, including three randomized controlled trials (RCTs)] with response rates ranging from 42 to 100% and included a meta-analysis, which showed that catatonic symptoms improved after ECT treatment. The number of ECT treatment sessions ranged from 3 to 35 with a frequency of 2–5 sessions/week, and the electrode placement was mostly bitemporal. The other two studies (38, 40) only provided qualitative descriptions, with response rates of 59%−100%, and concluded that ECT should be considered as first-line treatment for patients with catatonia and that early intervention with ECT should be encouraged to prevent unnecessary deterioration of patient's medical condition.

Two systematic reviews (37, 41) reported that ECT appeared to be well-tolerated and successful in treating catatonic schizophrenia, but only a few studies were included in these two reviews; specifically, one review (37) included four prospective case series, and the other (41) included three low-quality studies. The electrode placement for patients with catatonic schizophrenia was mostly bilateral; however, neither study described the treatment frequency. One of the two systematic reviews (41) reported that patients with catatonic schizophrenia responded faster to ECT than those with non-catatonic schizophrenia. Another review (37) pointed out that acute ECT was efficacious for late-life catatonic schizophrenia before and after relapse.

Two systematic reviews (32, 42) summarized the treatment effect for catatonia patients with ASD, and (31) one review (32) included 11 case studies, with a detailed description of the protocol. In these case studies, the number of treatment sessions ranged from 7 to 29 sessions, with a frequency ranging from three sessions/week to one session every 2–3 weeks, and the electrode placement was mostly bilateral. Almost all patients exhibited marked or dramatic improvement with ECT treatment, but the quality of the included studies was low; the authors concluded that there may have been an initial response to ECT, resulting in partial alleviation of catatonia symptoms, but this effect appeared to be temporary. Another review (42) included two retrospective longitudinal studies and did not describe the specific protocol; this review also emphasized that the use of short-term and maintenance ECT might be beneficial for catatonia patients with ASD. Overall, these two reviews revealed that maintenance ECT was necessary for sustained symptom alleviation.

Cronemeyer et al. (31) conducted a systematic review comparing the effectiveness of different treatment approaches for MC in terms of outcome and severity. This review included 23 ECT case reports; data synthesis was performed, and it was concluded that the most favorable outcome in MC patients was due to the administration of both benzodiazepines and ECT, with the highest rate of cases with full alleviation of symptoms (76.7%). Four systematic reviews described the effects of ECT treatment on catatonia in patients with COVID-19 (30), benzodiazepine withdrawal (39), OCD (35), and anti-NMDA receptor encephalitis (43). All included studies in the four reviews were case reports, with the number of patients ranging from 1 to 26, and the quality of the included studies was not evaluated. All four systematic reviews showed the effectiveness of ECT in alleviating catatonia symptoms, but most of the protocols included in the studies were not described in detail.

Døssing et al. (33) conducted a systematic review that included 23 retrospective studies and concluded that ECT had high treatment response rates for catatonia in children and adolescents. However, the treatment protocol was not described, and the quality assessment showed that the included studies were of low quality. Jaimes-Albornoz et al. (34) found that ECT was safe and effective in older patients, with the number of treatment sessions ranging from 2 to 25, mostly bilateral frontotemporal electrode placement, and a treatment frequency of two or three times a week. This review also highlights that medical risks, which may affect treatment outcomes, should be evaluated on an individual basis.

Overall, most reviews reported that ECT is effective for treating all forms of catatonia in patients of different ages, with a response rate of 42%−100%, and the treatment response rate was the highest in patients with catatonic schizophrenia and malignant catatonia. However, only one systematic review included a meta-analysis, and only five reviews assessed the evidence quality of the studies, resulting in low quality. Furthermore, most studies included in the reviews were case reports or observational studies; there were only three RCTs, which reduced the evidence quality. The number of studies included in the reviews ranged from 1 to 28. The number of treatment sessions ranged from 1 to 35, and seven studies (30, 31, 33, 35, 39, 41, 42) did not describe the treatment frequency or the site of the electrodes. Generally, ECT was administered three times per week on alternate days with bilateral electrode placement. Side effects such as mental confusion, cognitive/memory impairment, headache, and adverse effects associated with anesthesia were reported. Details of each review are provided in the Supplementary material.

Although most were case studies and RCTs were scarce, (1) these systematic reviews strongly indicated that ECT is effective for treating catatonia regardless of type or patient age; (2) ECT should be a first-line treatment for catatonia, i.e., not only initiated after ineffective pharmacotherapy but also as primary therapy; (3) maintenance ECT appeared to facilitate sustained improvements in catatonia symptoms; and (4) three sessions per week with bilateral electrode placement was the most common treatment with beneficial outcomes for catatonia patients.

### 3.2. rTMS for catatonia

A list of eligible studies is shown in Table 2. A total of 12 patients from 12 case reports were included. Seven of these patients (58.3%) were women. The average age of the patients was 38.9 years, with a range of 16–75 years; nine patients were adults, two patients were adolescents, and one patient did not have an age reported. Among the 12 case reports, six patients (50%) were diagnosed with schizophrenia, three were diagnosed with bipolar disorder (25%), one was diagnosed with depression, one was diagnosed with psychotic disorder, and one was diagnosed with organic catatonic disorder.

**Table 2:** Characteristics of the included rTMS studies.

**Study**	**Patient diagnosis**	**Sex**	**Age**	**Life stage**	**Previous ECT**	**Targeted brain region**	**Frequency**	**Intensity**	**Pulses**	**Number of sessions**	**Total pulses**	**Initial BFCRS score**	**Final BFCRS score**	**Symptom reduction**	**Improvement**	**Adverse effects**	**Reasons for receiving rTMS**
Grisaru et al. (47)	Schizophrenia	F	24	Adult	Considered	R dlPFC	20 Hz	80% MT	800 p/s	10	8,000	Not performed	Not performed	–	Rapid	Not described	Pharmacotherapy was ineffective
Saba et al. (54)	Schizophrenia	F	18	Adult	No	L dlPFC	10 Hz	80% MT	1,600 p/s	10	16,000	19	3	84%	Rapid	Not described	Not described
Trojak et al. (57)	Schizophrenia	M	45	Adult	1 session	L and R dlPFC and OFC (sequential)	10 Hz	110% MT	2,000 p/s	80	160,000	23	20	13%	Insufficient	Not described	Contraindication to ECT
Stip et al. (55)	Schizophrenia	M	Not described	Not described	556 sessions	Bilateral dlPFC	20 Hz	110% MT	3,000 p/s	108	32,400	31–46	< 15	61%	Sufficient, but variable	Not described	Safety concerns for ECT
Licht et al. (48)	Schizophrenia	F	55	Adult	Not described	SMA	1 Hz	66% MT	1,000 p/s	3	3,000	Not performed	Not performed	-	Rapid and long-lasting	Not described	Not described
Di Michele and Bolino (51)	Bipolar type I depression	F	75	Adult	Not described	L dlPFC	20 Hz	80% MT	400 p/s	7	2,800	Not performed	Not performed	-	Rapid and long-lasting	Not described	Pharmacotherapy was ineffective
Takamiya et al. (56)	Bipolar disorder	M	63	Adult	Yes	L dlPFC	10 Hz	120% MT	3,000 p/s	20	60,000	Not performed	Not performed	-	Rapid and long-lasting	No severe adverse effects	Contraindication to ECT
Marques et al. (53)	Bipolar type I medication-resistant depression	M	63	Adult	Refused	L dlPFC	iTBS	N/A	600 p/s, twice daily	30	18,000	23	7	70%	Rapid and long-term	Mild headache after the initial sessions	Refused ECT treatment
Kate et al. (49)	Refractory organic catatonia	F	22	Adult	Refused	Bilateral dlPFC	10 Hz (Day 1) 20 Hz (Days 2–10)	80% MT	800 p/s (Day 1) 1,600 p/s (Days 2–10)	10	14,800	32	9	72%	Rapid and long-lasting	Not described	Refused ECT
Ocampo et al. (52)	Psychotic disorder	F	30	Adult	No	Bilateral dlPFC (1st session) L dlPFC (2nd−10th sessions)	10 Hz (Day 1) 20 Hz (Days 2–10)	80% MT	800 p/s	10	8,000	Not performed	Not performed	-	Rapid and long-lasting	No adverse events	Pharmacotherapy was ineffective and ECT was not available
Marei and Rashed (50)	Depression	M	17	Adolescent	No	L dlPFC	N/A	45% MT	2,000 p/s	10	20,000	Not performed	Not performed	–	Rapid and long-lasting	Not described	Not described
Sharma et al. (46)	Schizophrenia	F	16	Adolescent	No	L dlPFC	10 Hz	100% MT	1,200 p/s	19	22,800	10	2	80%	Rapid and long-lasting alleviation	No adverse events	Not responsive to lorazepam, refused ECT

rTMS, repetitive transcranial magnetic stimulation; BFCRS, Bush-Francis Catatonia Rating Scale; F, female; M, male; dlPFC, dorsolateral prefrontal cortex; OFC, orbitofrontal cortex; L, left; R, right; MT, motor threshold; p/s, pulses per session; SMA, supplementary motor area; iTBS, intermittent theta burst stimulation.

Among these studies, nine patients (75%) received high-frequency (10-Hz or 20-Hz) stimulation over the dorsolateral prefrontal cortex (dlPFC), while one study applied low-frequency (1-Hz) rTMS over the supplementary motor area (SMA). In addition, Marques et al. reported administering intermittent theta burst stimulation (iTBS) over the left dlPFC and then 14 sessions of 10-Hz stimulation of the left dlPFC as maintenance treatment. Marei et al. did not report the frequency applied to the left dlPFC. The average number of pulses per day was 1,423, ranging from 400 to 3,000. The median number of treatment sessions was 10, ranging from 3 to 108, and the treatment intensity ranged from 45% of the motor threshold (MT) to 120% of the MT.

Except for one study (57), the remaining studies reported that rTMS successfully improved catatonia symptoms, with symptom reduction rates ranging from 61 to 84%. Among the 12 patients, eight did not describe adverse effects, three reported no adverse effects, and one reported mild headaches after the initial sessions. The reasons for rTMS selection were as follows: three patients chose rTMS because pharmacotherapy was ineffective, three patients refused ECT, two patients had contradictions to ECT, and one patient had safety concerns about ECT.

Most of the reported case studies applied rTMS as an intervention for catatonia in patients with schizophrenia. In addition, excitatory stimulation (high-frequency stimulation and iTBS) targeting the dlPFC was the most common treatment with beneficial outcomes for catatonia.

### 3.3. tDCS for catatonia

All the eligible studies describing tDCS interventions were case reports: one was a case series consisting of eight patients, and the remaining six studies were single case studies. Therefore, a total of 14 patients were included in this systematic review. The characteristics of the included reports are shown in Table 3. Among the 14 patients, eight were female patients (57.1%). The average age was 41.9 years, ranging from 14 to 65 years; except for one patient who was an adolescent, the remaining patients were adults. Most patients had schizophrenia spectrum disorder; specifically, one patient was diagnosed with ASD (64), 10 patients were diagnosed with schizophrenia, two patients were diagnosed with schizophrenia and ASD, and one had schizoaffective disorder.

**Table 3:** Characteristics of the included tDCS studies.

**Study**	**Patient diagnosis**	**Sex**	**Age**	**Life stage**	**Previous ECT**	**Targeted brain region**	**Intensity**	**Stimulationduration**	**Number of sessions**	**Initial BFCRS score**	**Final BFCRS score**	**Symptom reduction**	**Improvement**	**Adverse effects**	**Reasons for receiving tDCS**
Shiozawa et al. (65)	Schizophrenia	F	65	Adult	20 sessions	Anode over L dlPFC and cathode over R dlPFC	2 mA	20 min	10	32	3	91%	Rapid and long-term	Not described	ECT and pharmacotherapy were ineffective and rTMS was unavailable
Baldinger-Melich et al. (62)	Schizophrenia	M	42	Adult	15 sessions	Not described	2 mA	20 min	10	40–43	37	11%	Insufficient	Not described	ECT was ineffective
Chen et al. (63)	Schizophrenia	F	40	Adult	10 sessions	Anode over L dlPFC, cathode over R dlPFC	2 mA	20 min	10	7	3	57%	Rapid, but not long term	Not described	Pharmacotherapy was ineffective
Wysokiński (66)	Schizophrenia	F	58	Adult	17 sessions	Anode over L dlPFC and cathode over R dlPFC	2 mA	20 min	15	12	2	81%	Rapid	Mild side effects (mostly tingling and itching)	Treatment- refractory catatonia with bone fractures
Keeser et al. (61)	Schizophrenia and CCA	M	44	Adult	6 years	Anode over L dlPFC and cathode over R dlPFC	2 mA	20 min	330	69	4–12	88%	Improved	Not described	Drug treatment was ineffective
Haroche et al. (67)	Schizoaffective disorder	F	24	Adult	24 sessions	Anode over L dlPFC and cathode over L TPJ	2 mA	20 min	12	15	4	73%	Rapid and long-term	Not described	Treated unsuccessfully with lorazepam; ECT was stopped due to poor neurological tolerance and insufficient efficacy
Haroche et al. (67)	Schizophrenia	M	25	Adult	5 sessions	Anode over L dlPFC and cathode over L TPJ	2 mA	20 min	20	Not performed	Not performed	79%	Rapid	Not described	Treated unsuccessfully with lorazepam; ECT contraindications
Haroche et al. (67)	Schizophrenia and ASD	M	54	Adult	No	Anode over L dlPFC and cathode over L TPJ	2 mA	20 min	16	27	13	52%	Rapid and long-term	Not described	Treated unsuccessfully with lorazepam
Haroche et al. (67)	Schizophrenia	F	58	Adult	No	Anode over L dlPFC and cathode over L TPJ	2 mA	20 min	20	17	9	47%	Rapid	Not described	Treated unsuccessfully with lorazepam
Haroche et al. (67)	Schizophrenia	F	59	Adult	No	Anode over L dlPFC and cathode over L TPJ	2 mA	20 min	5	23	9	61%	Rapid	Not described	Treated unsuccessfully with lorazepam
Haroche et al. (67)	Schizophrenia and ASD	M	26	Adult	Considered, but not administered	Anode over L dlPFC and cathode over L TPJ	2 mA	20 min	10	24	17	29%	Rapid	Not described	Treated unsuccessfully with lorazepam
Haroche et al. (67)	Schizophrenia	M	54	Adult	4 courses	Anode over L dlPFC and cathode over L TPJ	2 mA	20 min	34	22	8	64%	Rapid	Not described	Treated unsuccessfully with lorazepam; ECT contraindications
Haroche et al. (67)	Schizophrenia	F	24	Adult	Refused	Anode over L dlPFC and cathode over L TPJ	2 mA	20 min	14	14	0	100%	Rapid	Not described	Treated unsuccessfully with lorazepam; refused ECT
Costanzo et al. (61)	ASD	F	14	Adolescent	Considered, but not administered due to safety concerns	Anode over L dlPFC and cathode over R dlPFC	1 mA	20 min	28	Not performed	Not performed	43%	Rapid and long-term	Not described	Drug-resistant catatonia and safety concerns for ECT

tDCS, transcranial direct current stimulation; BFCRS, Bush-Francis Catatonia Rating Scale; F, female; M, male; dlPFC, dorsolateral prefrontal cortex; L, left; R, right; ASD, autism spectrum disorder; CCA, complete corpus callosum agenesis; TPJ, temporoparietal junction.

The number of treatment sessions differed among patients, varying from 10 to 330. The stimulation duration in all studies was 20 min. One patient received 1-mA tDCS, and the remaining patients received 2-mA tDCS. Except for one study that did not report electrode placement, the remaining patients had the anode positioned over the left dlPFC, while the position of the cathode varied. The cathode was placed over the right dlPFC (in five patients) or the left temporoparietal junction (in eight patients).

Except for one study that reported insufficient improvement in catatonic symptoms, tDCS had a symptom reduction rate ranging from 29 to 100%. Most studies did not report adverse effects; one case reported mild side effects (mostly tingling and itching) (66), and another case series study consisting of eight patients reported only a burning sensation or tingling (67).

In general, studies on tDCS treatment of catatonia are scarce; however, those available suggest that tDCS is effective for treating catatonia. Most case reports administered 20-min sessions of 2-mA tDCS with the anode positioned over the left dlPFC. Almost all studies focused on catatonia in schizophrenia.

### 3.4. Results of rTMS and tDCS data analysis

Further analysis of BFCRS scores was performed for six patients who received rTMS and 12 patients who received tDCS. We used 15 as the post-treatment score for one case (55) because some studies reported BFCRS scores of <15 during rTMS. The paired-sample *t*-tests showed significant symptom improvement after rTMS (*t* = 4.489, *p* = 0.006). Because the BFCRS scores before and after tDCS were not normally distributed, we used the Wilcoxon signed-rank test to compare the tDCS-induced change in BFCRS scores in this group. There was a significant improvement in symptoms after tDCS (*z* = −3.065, *p* = 0.002). In addition, we compared the symptom reduction rate between the rTMS group and the tDCS group; these groups did not differ in the reduction rate (*t* = 0.038, *p* = 0.97). We also compared the symptom reduction rate of two cathode positions to determine whether different cathode placements resulted in different treatment responses. The results of the independent-sample *t*-test showed no significant difference (*t* = 0.297, *p* = 0.772).

### 3.5. Methodological quality/risk of bias assessment of the included research

The methodological quality of 14 systematic reviews based on the AMSTAR-2 tool is shown in Supplementary Table S1. One review (36) was considered “low quality,” while 13 reviews (30–35, 37–43) were considered “critically low quality.” None of the included reviews reported on the sources of funding for the included studies. In addition, the review considered “low quality” did not explain the selection of studies for inclusion in the review, provide a list of excluded studies, or justify the exclusions. Only five reviews (32, 33, 36, 41, 42) used a satisfactory technique for assessing the risk of bias in primary studies. A total of 11 reviews (30, 32–35, 37–41, 43) did not perform data synthesis. One study (42) performed a meta-analysis of the prevalence of catatonia in ASD patients; however, this review did not include data synthesis to calculate the efficacy of ECT treatment for patients with catatonia.

The risk of biased results of the rTMS case reports is demonstrated in Supplementary Table S2. Among the 12 rTMS case reports, 11 (46, 47, 49–57) showed a low risk of bias, and one (48) showed a moderate risk of bias. The overall scores for the rTMS case reports were as follows: 100% for two studies (46, 53), 87.5% for five studies (49, 52, 54, 56, 57), 75% for four studies (47, 50, 51, 55), and 50% for one study (48). Of all, eight (47–51, 54, 55, 57) of the 12 case reports did not describe adverse events (harms) or unanticipated events. One case report (48) did not clearly describe the patient's history, current clinical condition, diagnostic tests, or assessment methods and results.

The risk of biased results of the tDCS case studies and case series is demonstrated in Supplementary Table S2. All six tDCS case reports showed a low risk of bias, and the overall scores were as follows: 100% for one study (66), 87.5% for three studies (63–65), and 75% for two studies (61, 62). The tDCS case series conducted by Haroche et al. (67) showed a moderate risk of bias; in this case series, the criteria for inclusion and demographic information were not clearly described, and it was unclear whether patients were consecutively and completely included.

## 4. Discussion

This study is the first to assess three common NIBS techniques for catatonia treatment. We focused on clinical research evaluating their use in patients of varying ages and with different catatonia types. We found that ECT, rTMS, and tDCS were effective in treating catatonia. Our main findings were as follows: (1) *ECT should be administered as first-line treatment for catatonia and is effective for different types of catatonia and different ages of patients;* (2) *excitatory stimulation (high-frequency stimulation and iTBS) targeting the dlPFC was the most common rTMS protocol and was successful for treating catatonia*; and (3) *20-min sessions of 2-mA tDCS with the anode positioned over the left dlPFC were commonly used to treat catatonia in schizophrenia patients*. We also suggested that *early NIBS intervention may facilitate improvement in catatonia symptoms among patients with schizophrenia and using rTMS or tDCS to maintain symptom improvements may be advantageous*. Finally, there is a need for future high-quality RCTs with improved designs to determine the efficacy and optimal schedule of NIBS treatments for patients with catatonia.

### 4.1. ECT

We first summarized the efficacy of ECT treatment for all catatonia types regardless of etiology and showed that ECT can be used in children, adolescents, and elderly patients with catatonia. The guidelines of the American Psychiatric Association (APA) have indicated that ECT is the most effective treatment for catatonic syndrome (70). Our findings supported this recommendation and extended it to different etiologies of catatonia and different age groups of patients. The review by Benarous et al. also found that ECT was as effective in children or adolescents with catatonia as it was in adults (71). These findings were consistent with our study and supported the use of ECT in children and adolescents with catatonia.

Notably, ECT should be considered the first-line treatment for catatonia, not only after a lack of response to pharmacotherapy but also as the primary therapy. In clinical practice, benzodiazepines are the most common intervention administered to patients with catatonia. For patients with life-threatening conditions or treatment-resistant patients, ECT is the treatment of choice. Previous studies have proposed that ECT should be administered to patients with catatonia who are unresponsive to benzodiazepines or to patients with life-threatening conditions (3, 72). Our findings extended these recommendations, suggesting that ECT can be initiated in other patients, such as patients with catatonia and ASD or schizophrenia, both of which may respond poorly to benzodiazepine treatment. ECT is used as a secondary treatment in most patients due to its association with potential side effects, stigma, and inability to be administered in all settings. However, early ECT intervention can prevent undue deterioration of the patient's medical condition. Hence, it is vital to determine whether ECT is necessary and to administer it as soon as possible. Indeed, we emphasized this finding as it merits further clinical research to attract the attention and recognition of more clinicians.

In addition to being the first-line intervention of choice, ECT can also maintain symptomatic remission. We suggested that maintenance ECT is beneficial and essential for sustaining symptomatic remission and should be recommended in future clinical practice. The frequency of maintenance ECT ranged from three times per week to once every 2–3 weeks, and studies have reported rapid recurrence of symptoms after suspension or discontinuation of ECT (32). Furthermore, among catatonia patients previously responsive to ECT, this treatment could be applied in relapses (37).

### 4.2. rTMS

This systematic review found that most studies of catatonia treated with rTMS reported symptom improvement; further analysis suggested significant improvement in catatonia symptoms after rTMS. This finding is consistent with those of previous studies (29, 58); our review further included additional studies that showed that rTMS was beneficial for treating various subtypes of catatonia, such as catatonic schizophrenia, catatonia associated with mood disorders, and organic catatonia. However, all studies included in this review were case reports, which reduce the quality of evidence. In the future, RCTs are needed to confirm the efficacy and safety of rTMS for catatonia.

Different protocols have been used in studies of rTMS. Excitatory stimulation (high-frequency stimulation and iTBS) targeting the dlPFC was the most common method that successfully treated catatonia; the dlPFC is also a common stimulation target for treating psychiatric disorders and decreased dlPFC activity has been linked to catatonia (73). In addition, one study administered low-frequency stimulation to the SMA, resulting in rapid symptom improvement (11). These findings demonstrate that rTMS is a promising intervention for catatonia with the potential to specifically modulate the neural mechanism underlying catatonia.

Due to the scarcity of research, we did not compare the treatment efficacy of different protocols. In addition, the types of catatonia treated with rTMS in this review varied. The most common diagnosis was schizophrenia, followed by bipolar disorder. Hence, further evidence is needed to determine whether different etiologies of catatonia require different treatment modalities. Furthermore, two adolescent patients diagnosed with catatonia were successfully treated with rTMS and reported no adverse effects. Therefore, rTMS may be an effective and safe intervention for adolescents with catatonia, but further studies are needed.

### 4.3. tDCS

Additionally, tDCS appeared to successfully treat catatonia, even though we were only able to include relevant case studies. Most patients treated with tDCS were diagnosed with schizophrenia, and their BFCRS scores showed improvements in catatonia symptoms. These findings demonstrate the benefits of tDCS in schizophrenia patients with catatonic symptoms. However, one case showed insufficient improvement in catatonia symptoms after tDCS; this patient was also unresponsive to ECT (62). For patients who are refractory to pharmacotherapy as well as ECT and tDCS, deep brain stimulation may be helpful; however, further research is needed to verify this theory. One case study reported the successful use of tDCS for an adolescent with ASD and catatonia (64). In addition, the catatonia symptoms of another two patients diagnosed with schizophrenia and ASD also improved after tDCS (67).

The tDCS protocols were similar, typically involving a 20-min session of 2-mA tDCS with the anode positioned over the left dlPFC. Combining these findings with fMRI data (61), we speculate that the mechanism by which anodal tDCS over the left dlPFC reduces catatonia symptoms may be through modulation of the connectivity between prefrontal regions and other regions. Additionally, two cathodal positions (over the right dlPFC and left TPJ) were identified in this review. However, further analysis did not yield a difference in treatment efficacy between the two cathodal positions. Thus, optimal tDCS protocols should be investigated in future studies as the number of cases is low.

Most studies administered tDCS intervention in the acute phase of catatonia, applying 10–34 sessions. However, one study included 330 sessions over almost 4 years and showed improvement in catatonia symptoms (61), indicating that tDCS could be used as a long-term maintenance treatment for catatonia. In addition, another case also underwent maintenance tDCS (two sessions every 2 weeks) after an initial improvement in catatonia symptoms, resulting in long-term clinical stability. Overall, maintenance treatment with tDCS may facilitate long-term improvement in catatonia symptoms.

### 4.4. Methodological quality/risk of bias assessment of the included research

All systematic reviews of ECT included in this review were classified as having “low quality” or “critically low quality” according to the AMSTAR-2 critical appraisal criteria. The methodological quality of the systematic reviews was limited by the lack of the following: registration and funding data, a comprehensive search strategy, a list and justification of excluded articles, a detailed description of the included studies, an assessment of the potential impact of risk of bias, and an explanation of the risk of bias. Future research should consider the abovementioned issues. Only one systematic review (36) included a meta-analysis to explore the efficacy of ECT treatment for catatonia; the scarcity of meta-analyses may be due to the limited RCT studies and the various etiologies of catatonia and various ECT protocols in the studies. While the risk of bias for the included rTMS and tDCS studies was low, the included articles were all case studies. Haroche et al. (67) reported a tDCS case series of eight patients; however, this case series showed a moderate risk of bias. Future case series should follow the Case Report (CARE) guidelines (74) which provide a checklist that could assist researchers in publishing complete and meaningful clinical information. In general, high-quality RCTs with large sample sizes are necessary to demonstrate the clinical effectiveness of NIBS for catatonia.

### 4.5. NIBS techniques to treat catatonia

Given the evidence above, we recommend applying ECT as the first-line treatment for catatonia; rTMS and tDCS could be alternative interventions for catatonia or supplement treatment with ECT and pharmacotherapy. Our review demonstrated that using rTMS or tDCS to maintain and consolidate symptom alleviation may be advantageous. On the one hand, maintenance treatment is important for catatonia recovery; however, ECT may not be available for use as long-term maintenance therapy, especially in outpatients. On the other hand, adverse effects are an important factor in treatment safety, as ECT has potential adverse effects, especially cognitive impairment. rTMS and tDCS are relatively safe treatment modalities without the risk of dependency or the need for a general anesthetic. Based on these limited data, the side effects of rTMS and tDCS for the treatment of catatonia were milder than those of ECT, indicating that rTMS and tDCS may be better treatment options than ECT due to their safety. Overall, the adverse effects of treatment have not been adequately addressed, and the safety of NIBS in patients with catatonia should be investigated in the future.

At present, NIBS techniques play increasingly crucial roles in the treatment of psychiatric disorders, especially in treatment-resistant diseases. Flexible and convenient NIBS techniques, such as rTMS and tDCS, should be actively applied to manage the entire course of mental illness. Furthermore, clinicians should know the optimal treatment protocol, and professionals should increase the publicity and popularization of NIBS techniques. Moreover, most studies included in this review focused on adult patients; fewer studies have focused on children and adolescents, although these studies have reported a great response to NIBS treatment. Based on these limited studies, we support the use of NIBS in children and adolescents; however, further studies in children and adolescents are needed to elucidate the efficacy of NIBS treatment of catatonia, especially tDCS and rTMS.

### 4.6. Limitations

In this review, we evaluated the methodological quality of the included SRs by using the AMSTAR-2 tool and assessed the risk of bias of the included case studies by using the Joanna Briggs Institute Critical Appraisal tools; however, the included systematic reviews of ECT were classified as having “low quality” or “critically low quality.” The overall risk of bias in the rTMS and tDCS studies was low; however, all the studies were case studies. In addition, most of the included studies in the ECT systematic review were observational studies or case reports; thus, high-quality RCTs are needed to verify the treatment efficacy of NIBS techniques and determine optimal protocols. Second, the low number of publications limited the comparison of the effectiveness of NIBS techniques for different types of catatonia.

## 5. Conclusion

We recommend that ECT be used as a first-line treatment for patients with catatonia. rTMS and tDCS could represent optional treatments for catatonia in the future since new RCTs improve their efficacy. Future high-quality RCTs are crucial to validate the efficacy of NIBS techniques and to confirm further optimal NIBS protocols for treating different subtypes of catatonia. NIBS techniques represent promising therapies for catatonia.

## Data availability statement

The original contributions presented in the study are included in the article/Supplementary material, further inquiries can be directed to the corresponding author.

## Author contributions

HX and YM: conceptualization, methodology, writing—original draft, writing—reviewing and editing, and visualization. SL: conceptualization, methodology, and writing—original draft. YC and HS: methodology and writing—original draft, writing—reviewing and editing. GD, MW, and YZ: methodology. CQ: conceptualization, methodology, writing—original draft, writing—reviewing and editing, and supervision. All authors contributed to the article and approved the submitted version.
