# Supplementary material for: Non-invasive brain stimulation for treating catatonia: a systematic review
Source: Front Psychiatry. 2023 May 16;14:1135583. doi: 10.3389/fpsyt.2023.1135583 (PMC10227525; doi:10.3389/fpsyt.2023.1135583)
Supplement: Supplementary file 1 [file Data_Sheet_1.docx]

**Noninvasive Brain Stimulation for Catatonia:**

**A Systematic Review**

**Supplemental materials:**

**2.1 Search Strategy**

For identifying articles on rTMS treatment for catatonia, numerous combinations of words were used for each database, including“Schizophrenia, Catatonic” OR “Catatonic Schizophrenias” OR “Schizophrenias, Catatonic” OR “Catatonic Schizophrenia” OR “Catatonia” OR “Catatonias” OR “Catatonia, Organic” OR “Catatonias, Organic” OR “Organic Catatonia” OR “Organic Catatonias” OR “Organic Catatonic Disorder” OR “Catatonic Disorder, Organic” OR “Catatonic Disorders, Organic” OR “Organic Catatonic Disorders” OR “Schizophreniform Catatonia” OR “Catatonia, Schizophreniform” OR “Catatonias, Schizophreniform” OR ‘Schizophreniform Catatonias” OR “Catatonia, Malignant” OR “Catatonias, Malignant” OR “Malignant Catatonia” OR “Malignant Catatonias” OR “Lethal Catatonia” OR “Catatonia, Lethal” OR “Catatonias, Lethal” OR “Lethal Catatonias” AND “Transcranial Magnetic Stimulation” OR “Magnetic Stimulation, Transcranial” OR “Magnetic Stimulations, Transcranial” OR “Stimulation, Transcranial Magnetic” OR “Stimulations, Transcranial Magnetic” OR “Transcranial Magnetic Stimulations” OR “Transcranial Magnetic Stimulation, Single Pulse” OR “Transcranial Magnetic Stimulation, Paired Pulse” OR “Transcranial Magnetic Stimulation, Repetitive”.

For identifying articles on tDCS treatment for catatonia, numerous combinations of words were used for each database, including“Schizophrenia, Catatonic” OR “Catatonic Schizophrenias” OR “Schizophrenias, Catatonic” OR “Catatonic Schizophrenia” OR “Catatonia” OR “Catatonias” OR “Catatonia, Organic” OR “Catatonias, Organic” OR “Organic Catatonia” OR “Organic Catatonias” OR “Organic Catatonic Disorder” OR “Catatonic Disorder, Organic” OR “Catatonic Disorders, Organic” OR “Organic Catatonic Disorders” OR “Schizophreniform Catatonia” OR “Catatonia, Schizophreniform” OR “Catatonias, Schizophreniform” OR ‘Schizophreniform Catatonias” OR “Catatonia, Malignant” OR “Catatonias, Malignant” OR “Malignant Catatonia” OR “Malignant Catatonias” OR “Lethal Catatonia” OR “Catatonia, Lethal” OR “Catatonias, Lethal” OR “Lethal Catatonias” AND “Transcranial Direct Current Stimulation” OR “tDCS” OR “Cathodal Stimulation Transcranial Direct Current Stimulation” OR “Cathodal Stimulation tDCS” OR “Cathodal Stimulation tDCSs**”** OR “Stimulation tDCS, Cathodal” OR “Stimulation tDCSs, Cathodal” OR “tDCS, Cathodal Stimulation” OR “tDCSs, Cathodal Stimulation” OR “Transcranial Random Noise Stimulation” OR “Transcranial Alternating Current Stimulation” OR “Transcranial Electrical Stimulation” OR “Electrical Stimulation, Transcranial” OR “Electrical Stimulations, Transcranial” OR “Stimulation, Transcranial Electrical” OR “Stimulations, Transcranial Electrical” OR “Transcranial Electrical Stimulations” OR “Anodal Stimulation Transcranial Direct Current Stimulation” OR “Anodal Stimulation tDCS” OR “Anodal Stimulation tDCSs” OR “Stimulation tDCS, Anodal” OR “Stimulation tDCSs, Anodal” OR “tDCS, Anodal Stimulation” OR “tDCSs, Anodal Stimulation” OR “Repetitive Transcranial Electrical Stimulation”.

For identifying reviews on ECT treatment for catatonia, numerous combinations of words were used for each database, including“Schizophrenia, Catatonic” OR “Catatonic Schizophrenias” OR “Schizophrenias, Catatonic” OR “Catatonic Schizophrenia” OR “Catatonia” OR “Catatonias” OR “Catatonia, Organic” OR “Catatonias, Organic” OR “Organic Catatonia” OR “Organic Catatonias” OR “Organic Catatonic Disorder” OR “Catatonic Disorder, Organic” OR “Catatonic Disorders, Organic” OR “Organic Catatonic Disorders” OR “Schizophreniform Catatonia” OR “Catatonia, Schizophreniform” OR “Catatonias, Schizophreniform” OR ‘Schizophreniform Catatonias” OR “Catatonia, Malignant” OR “Catatonias, Malignant” OR “Malignant Catatonia” OR “Malignant Catatonias” OR “Lethal Catatonia” OR “Catatonia, Lethal” OR “Catatonias, Lethal” OR “Lethal Catatonias” AND “Electroconvulsive Therapy” OR “Electroconvulsive Therapies” OR “Therapies, Electroconvulsive” OR “Therapy, Electroconvulsive” OR “Electroshock Therapy” OR “Electroshock Therapies” OR “Therapies, Electroshock” OR “Therapy, Electroshock” OR “Convulsive Therapy, Electric” OR “Convulsive Therapies, Electric” OR “Electric Convulsive Therapies” OR “Electric Convulsive Therapy” OR “Therapies, Electric Convulsive“ OR “Therapy, Electric Convulsive” OR “Shock Therapy, Electric” OR “Electric Shock Therapies” OR “Electric Shock Therapy“ OR “Shock Therapies, Electric“ OR “Therapies, Electric Shock” OR “Therapy, Electric Shock” OR “ECT (Psychotherapy)” AND “Systematic Review” OR “Meta-Analysis” OR “meta” OR “Mata-Analytic Review” OR “review”.

**3.1 ECT for Catatonia**

Notably, Leroy et al. [1] conducted the first and only meta-analysis to examine the association between catatonia and ECT across 28 studies (3 randomized controlled trials and one case series nested in an RCT, 12 prospective case series, and 12 retrospective case series) with 564 patients. In this review, the mean age range was 15.95-61.5 years, and specifically, 83% of the studies showed a female predominance. The number of ECT treatment sessions ranged from 3 to 35, with a frequency of 2-5 sessions/week (11 studies performed 3/week), and the electrode placement was mostly bitemporal but bifrontal, bilateral frontotemporal, bilateral temporoparietal, and unilateral placements were also found; ECT intensity was assessed by the titration method, age or half-age method and/or with a fixed intensity. These studies showed that the response rates of ECT treatment in patients with catatonia ranged from 42 to 100%. In addition, ten studies with a total of 211 participants (standard mean difference (SMD)=-3.14, 95% CI [-3.95; -2.34]) were included in the quantitative analysis (meta-analysis) and showed that catatonic symptoms improved after ECT treatment, and seven studies reported adverse effects, including mental confusion, headache, and memory loss, or adverse effects associated with anesthesia. However, the heterogeneity was high (I2=76.6%, Q test: p<0.001), and the quality of the RCTs was low. The analysis failed to demonstrate the efficacy and protocols of ECT treatment in catatonia patients.

Two other noteworthy systematic reviews also investigated the effectiveness of ECT treatment for patients diagnosed with catatonia [2, 3]. Luchini et al. presented a systematic review of 8 open observational studies with different diagnoses (mood disorder, psychotic disorder, organic mental disorder, and organic mental disorder) that included at least 10 patients who were treated with ECT for catatonia symptoms and reported a response rate ranging from 80% to 100%. The number of ECT treatment sessions ranged from 12 to 20 with a frequency of 3 sessions/week, and the electrode placement was mostly bitemporal (four studies). The half-age method was the most commonly used method to determine the intensity of stimulation and to measure the length of the seizure by electroencephalogram (EEG), which should be at least 25 seconds. The authors summarized that ECT was effective in patients with all forms of catatonia included in this systematic review and should be considered as a first-line treatment in patients with malignant catatonia (MC), neuroleptic malignant syndrome (NMS), delirious mania or severe catatonic excitement and in all catatonic patients who are refractory/partially responsive to benzodiazepines. Moreover, early ECT intervention was encouraged to avoid undue deterioration of a patient’s medical condition.

Pelzer et al. [3] also conducted a systematic review that included eleven studies describing the efficacy of ECT as a treatment method for catatonia. Among the 11 studies (2 prospective cohort studies, 5 retrospective cohort studies, 1 RCT), the number of ECT treatment sessions ranged from 2 to 13 with a frequency of 3 sessions/week, and the electrode placement was mostly bilateral. In six studies, ECT was performed as a secondary therapy when there was no or an insufficient response to benzodiazepines. ECT was initiated after ineffective pharmacotherapy and as a primary therapy, such as in life-threatening situations. The percentages, represented in terms of response and remission, ranged from 59% to 100%. In six studies, side effects such as cognitive/memory impairment or headache were actively reported. The authors concluded that ECT was a very effective therapy for catatonia, even when benzodiazepine (lorazepam) treatment failed. In addition, ECT may be a good alternative to pharmacotherapy in catatonia patients with life-threatening situations.

There were also some special reviews about ECT for catatonia patients, detailed as follows:

a. Liu et al [4] investigated the effectiveness of ECT in patients with **late-life catatonic schizophrenia**. They identified four prospective case series involving middle-aged (49-64 years old) and older participants with catatonic schizophrenia. The electrode placement was bilateral, and the number of treatment sessions in the two studies was twelve, while the other two studies did not describe the number of treatment session. This systematic review summarized that ECT appeared to be well tolerated and successfully treated older patients with catatonic schizophrenia. In addition, ECT was shown to be efficacious when catatonia relapsed after ECT treatment. **Bilateral acute and maintenance ECT treatment appear to be effective in older patients with schizophrenia.**

b. Pompili et al. [5] identified three articles (1 cohort study, 1 observational study, 1 prospective study) regarding patients with **catatonic schizophrenia** treated with ECT. The mean number of sessions was 8.4, and the most common reason for the use of ECT was to augment pharmacotherapy. This review compared the treatment efficacy of ECT for different types of schizophrenia and showed that patients with catatonia schizophrenia responded significantly better to ECT than patients with any other subtype of schizophrenia. The authors concluded that **ECT should be recommended for schizophrenia patients with catatonia**.

c. Cronemeyer et al. [6] conducted a case series analysis of 117 **malignant catatonia** case reports, which compared the remission of patients who received different treatments for malignant catatonia. Full remission rates were significantly higher and death rates were significantly lower in the ‘BZD and ECT’ group than in the other therapy groups. They concluded that **malignant catatonia patients who were treated with both benzodiazepines and ECT showed the most favorable outcomes**.

d. Jaimes-Albornoz et al. [7] conducted a systematic review of case studies for **catatonia in OCD** patients, which included 16 OCD patients with catatonia. The number of ECT treatment sessions ranged from 8 to 21. Ten of the 16 patients received ECT, and four of them achieved complete resolution of catatonia. This review highlighted the importance of treating the underlying etiology of catatonia, and **the treatment of the etiology of catatonia led to resolution more frequently than symptomatic treatment alone**.

e. Oldham et al. [8] included a patient with **benzodiazepine withdrawal catatonia** treated with ECT with a good effect and concluded that withdrawal catatonia was responsive to ECT.

f. Warren et al. [9] examined the efficacy of ECT for NMDA receptor encephalitis, including 25 studies of 26 **NMDA receptor encephalitis patients with catatonia** symptoms treated with ECT. The number of ECT treatment sessions ranged from 1 to 33, and the electrode placement was bilateral temporal in two studies, bilateral in one study, and not described in 22 studies. After ECT treatment, the symptoms of five (29.4%) of the 26 patients were resolved, and eight patients (47.1%) had improved catatonia symptoms. This review suggested that **ECT appeared to be an effective and safe adjuvant treatment in anti-NMDA receptor encephalitis patients, particularly for those with catatonia symptoms**.

g. Austgen et al. [10] conducted a systematic review of the use of ECT in patients with neuropsychiatric complications from COVID-19 and reported a case in which ECT was used to achieve remission in a patient who developed new-onset, treatment-resistant depression, psychosis, and **catatonia associated with COVID-19**. One of the three articles identified in this study also reported a patient diagnosed with COVID-19 and catatonia who was successfully treated with ECT. The number of treatment sessions ranged from 9 to 10.

h. DeJong et al. [11] examined the effect of interventions used to treat **catatonic symptoms in people with ASD**, which included 12 patients with ages ranging from 14 to 19 years. The number of treatment sessions ranged from 7 to 29, the treatment frequency ranged from 3 sessions/week to one session every 2-3 weeks, the electrode placement was bilateral in nine studies, and two studies used bilateral or unilateral electrode placement. Almost all patients reported a marked or dramatic improvement after ECT, but the effect appeared to be temporary. Most studies included in this review made no reference to any adverse effects of ECT treatment. In addition, the standard of the studies included in this systematic review was poor. Hence, the authors concluded that **there may be an initial response to ECT treatment, which is temporary, and maintenance ECT seems to be needed to sustain any benefit.**

i. Vaquerizo-Serrano [12] et al. also investigated the treatment efficacy of ECT for **ASD patients with catatonia**, which included 2 retrospective studies of patients who were treated with ECT. This review showed that ECT improved catatonia symptoms, but benzodiazepines did not show a specific benefit in the resolution of catatonia. **The maintenance of electroconvulsive therapy was necessary for sustaining symptom remission**.

j. Døssing and Pagsberg [13] conducted a systematic review regarding the efficacy of ECT in **children and adolescents**. Twenty-three studies (2 retrospective studies, 6 case series, 15 case studies) of ECT treatment with a total of 78 catatonia patients ranging from 6 to 19 years of age were identified. Similarly, the evidence quality of the identified studies was low. They found no absolute contraindications for ECT in children and adolescents, and the patients had a high treatment response, suggesting that ECT could be considered for treating catatonia in children and adolescents.

k. Jaimes-Albornoz et al. [14] conducted a systematic review of **catatonia in** **elderly individuals**. The number of treatment sessions ranged from 2 to 25, the electrode placement in most studies was bilateral frontotemporal, and the treatment frequency was 2 or 3 times a week. The authors reported that ECT was safe and effective in the treatment of catatonia in elderly patients. Considering that three elderly patients with catatonia related to general medical conditions had only a partial response to ECT after 7-8 treatments, the authors suggested that medical risk must be evaluated separately.

Table S1**:** The appraisal of risk of bias for systematic reviews and meta-analyses of ECT using the AMSTAR-2 tool

| AMSTAR-2 question^*^ | 1 | 2 | 3 | 4 | 5 | 6 | 7 | 8 | 9 | 10 | 11 | 12 | 13 | 14 | 15 | 16 | Overall quality |
| --- | --- | --- | --- | --- | --- | --- | --- | --- | --- | --- | --- | --- | --- | --- | --- | --- | --- |
| Study |  |  |  |  |  |  |  |  |  |  |  |  |  |  |  |  |  |
| Leroy,  et al., 2018 | Y | Y | N | Y | Y | Y | N | Y | Y | N | Y | Y | Y | Y | Y | Y | Low |
| Luchini,  et al., 2015 | N | N | Y | N | Y | N | N | Y | N | N | NA | NA | N | N | NA | N | Critically Low |
| Pelzer et al., 2018 | N | N | N | PY | Y | N | N | Y | N | N | NA | NA | N | N | NA | Y | Critically Low |
| Liu et al., 2014 | N | N | N | PY | N | N | N | N | N | N | NA | NA | N | N | NA | Y | Critically Low |
| Pompili et al., 2013 | N | N | N | PY | Y | Y | N | N | Y | N | NA | NA | N | N | NA | Y | Critically Low |
| Cronemeyer et al., 2022 | Y | N | N | PY | Y | N | N | N | N | N | N | N | N | N | N | Y | Critically Low |
| DeJong et al., 2014 | N | N | N | PY | N | N | N | PY | Y | N | NA | NA | N | N | NA | Y | Critically Low |
| Vaquerizo-Serrano et al., 2021 | Y | Y | N | Y | Y | Y | N | N | Y | N | N | N | N | N | N | Y | Critically Low |
| Jaimes-Albornoz et al., 2020 | N | N | N | PY | N | N | N | PY | N | N | NA | NA | N | N | NA | Y | Critically Low |
| Austgen et al., 2022 | N | N | N | PY | Y | N | N | PY | N | N | NA | NA | N | N | NA | Y | Critically Low |
| Oldham and Desan, 2016 | N | N | Y | PY | N | N | N | PY | N | N | NA | NA | N | N | NA | Y | Critically Low |
| Warren et al., 2019 | Y | Y | Y | PY | Y | N | N | Y | N | N | NA | NA | N | N | NA | Y | Critically Low |
| Døssing et al., 2021 | N | N | N | PY | N | N | N | PY | Y | N | NA | NA | N | N | NA | N | Critically Low |
| Jaimes-Albornoz et al., 2022 | N | N | N | PY | Y | N | N | PY | N | N | NA | NA | N | N | NA | Y | Critically Low |

Abbreviations: Yes (Y), No (N), Partial Yes (PY), Not applicable (NA).

**^*^ AMSTAR-2 questions:**

1. Did the research questions and inclusion criteria for the review include the components of PICO?

2. Did the report of the review contain an explicit statement that the review methods were established prior to the conduct of the review and did the report justify any significant deviations from the protocol?

3. Did the review authors explain their selection of the study designs for inclusion in the review?

4. Did the review authors use a comprehensive literature search strategy?

5. Did the review authors perform study selection in duplicate?

6. Did the review authors perform data extraction in duplicate?

7. Did the review authors provide a list of excluded studies and justify the exclusions?

8. Did the review authors describe the included studies in adequate detail?

9. Did the review authors use a satisfactory technique for assessing the risk of bias (RoB) in individual studies that were included in the review?

10. Did the review authors report on the sources of funding for the studies included in the review?

11. If meta-analysis was performed did the review authors use appropriate methods for statistical combination of results?

12. If meta-analysis was performed, did the review authors assess the potential impact of RoB in individual studies on the results of the meta-analysis or other evidence synthesis?

13. Did the review authors account for RoB in individual studies when interpreting/discussing the results of the review?

14. Did the review authors provide a satisfactory explanation for, and discussion of, any heterogeneity observed in the results of the review?

15. If they performed quantitative synthesis did the review authors carry out an adequate investigation of publication bias (small study bias) and discuss its likely impact on the results of the review?

16. Did the review authors report any potential sources of conflict of interest, including any funding they received for conducting the review?

Table S2 The appraisal of risk of bias for rTMS studies using the Joanna Briggs Institute critical appraisal checklist for case reports.

| JBI  question | Q1 | Q2 | Q3: | Q4 | Q5 | Q6 | Q7 | Q8 | Bias  Risk |
| --- | --- | --- | --- | --- | --- | --- | --- | --- | --- |
| Study |  |  |  |  |  |  |  |  |  |
| Grisaru et al., 1998 | Y | Y | Y | N | Y | Y | N | Y | Low |
| Saba et al., 2002 | Y | Y | Y | Y | Y | Y | N | Y | Low |
| Trojak et al., 2014 | Y | Y | Y | Y | Y | Y | N | Y | Low |
| Stip et al., 2017 | U | Y | Y | Y | Y | Y | N | Y | Low |
| Licht et al., 2021 | Y | U | U | U | Y | Y | N | Y | Moderate |
| Di Michele and Bolino, 2006 | Y | Y | Y | U | Y | Y | N | Y | Low |
| Takamiya et al., 2015 | Y | Y | Y | U | Y | Y | Y | Y | Low |
| Marques et al., 2021 | Y | Y | Y | Y | Y | Y | Y | Y | Low |
| Kate et al., 2011 | Y | Y | Y | Y | Y | Y | N | Y | Low |
| Ocampo et al., 2022 | Y | Y | Y | U | Y | Y | Y | Y | Low |
| Marei and Rashed, 2017 | Y | Y | Y | Y | U | Y | N | Y | Low |
| Sharma et al., 2018 | Y | Y | Y | Y | Y | Y | Y | Y | Low |

Abbreviation: Yes (Y); No (N); Unclear (U); Not applicable (NA).

Q1: Were patient’s demographic characteristics clearly described?

Q2: Was the patient’s history clearly described and presented as a timeline?

Q3: Was the current clinical condition of the patient on presentation clearly described?

Q4: Were diagnostic tests or assessment methods and the results clearly described?

 Q5: Was the intervention(s) or treatment procedure(s) clearly described?

Q6: Was the post-intervention clinical condition clearly described?

Q7: Were adverse events (harms) or unanticipated events identified and described?

Q8: Does the case report provide takeaway lessons?

Table S3 The appraisal of risk of bias for tDCS studies using the Joanna Briggs Institute critical appraisal checklist for case reports and case series

| Study design | JBI  question | Q1 | Q2 | Q3: | Q4 | Q5 | Q6 | Q7 | Q8 | Q9 | Q10 | Bias  Risk |
| --- | --- | --- | --- | --- | --- | --- | --- | --- | --- | --- | --- | --- |
| Case report | Study |  |  |  |  |  |  |  |  |  |  |  |
|  | Shiozawa et al., 2013 | Y | Y | Y | Y | Y | Y | N | Y | - | - | Low |
|  | Baldinger-Melich et al., 2016 | Y | Y | Y | Y | U | Y | N | Y | - | - | Low |
|  | Chen et al., 2018 | Y | Y | Y | Y | Y | Y | N | Y | - | - | Low |
|  | Wysokiński, A. (2020） | Y | Y | Y | Y | Y | Y | Y | Y | - | - | Low |
|  | Keeser et al., 2017 | Y | Y | U | Y | Y | Y | N | Y | - | - | Low |
|  | Costanzo et al., 2015] | Y | Y | Y | Y | Y | Y | N | Y | - | - | Low |
| Case series | Haroche et al., 2022 | N | Y | Y | U | U | Y | Y | Y | N | Y | Moderate |

Abbreviation: Yes (Y); No (N); Unclear (U); Not applicable (NA).

***Case report***

Q1: Were patient’s demographic characteristics clearly described?

Q2: Was the patient’s history clearly described and presented as a timeline?

Q3: Was the current clinical condition of the patient on presentation clearly described?

Q4: Were diagnostic tests or assessment methods and the results clearly described?

 Q5: Was the intervention(s) or treatment procedure(s) clearly described?

Q6: Was the post-intervention clinical condition clearly described?

Q7: Were adverse events (harms) or unanticipated events identified and described?

Q8: Does the case report provide takeaway lessons?

***Case series***

Q1: Were there clear criteria for inclusion in the case series?

Q2: Was the condition measured in a standard, reliable way for all participants included in the case series?

Q3: Were valid methods used for identification of the condition for all participants included in the case series?

Q4: Did the case series have consecutive inclusion of participants?

Q5: Did the case series have complete inclusion of participants?

Q6: Was there clear reporting of the demographics of the participants in the study?

Q7: Was there clear reporting of clinical information of the participants?

Q8: Were the outcomes or follow-up results of cases clearly reported?

Q9: Was there clear reporting of the presenting site(s)/clinic(s) demographic information?

Q10: Was statistical analysis appropriate?

**References:**

1. Leroy, A., et al., *Is electroconvulsive therapy an evidence-based treatment for catatonia? A systematic review and meta-analysis.* Eur Arch Psychiatry Clin Neurosci, 2018. **268**(7): p. 675-687.

2. Luchini, F., et al., *Electroconvulsive therapy in catatonic patients: Efficacy and predictors of response.* World journal of psychiatry, 2015. **5**(2): p. 182-92.

3. Pelzer, A.C., F.M. van der Heijden, and E. den Boer, *Systematic review of catatonia treatment.* Neuropsychiatr Dis Treat, 2018. **14**: p. 317-326.

4. Liu, A.Y., et al., *Brain stimulation in the treatment of late-life severe mental illness other than unipolar nonpsychotic depression.* American Journal of Geriatric Psychiatry, 2014. **22**(3): p. 216-240.

5. Pompili, M., et al., *Indications for electroconvulsive treatment in schizophrenia: A systematic review.* Schizophrenia Research, 2013. **146**(1-3): p. 1-9.

6. Cronemeyer, M., et al., *Malignant catatonia: Severity, treatment and outcome–a systematic case series analysis.* World Journal of Biological Psychiatry, 2022. **23**(1): p. 78-86.

7. Jaimes-Albornoz, W., et al., *Catatonia in obsessive-compulsive disorder: A systematic review of case studies.* Asian Journal of Psychiatry, 2020. **54**.

8. Oldham, M.A. and P.H. Desan, *Alcohol and Sedative-Hypnotic Withdrawal Catatonia: Two Case Reports, Systematic Literature Review, and Suggestion of a Potential Relationship With Alcohol Withdrawal Delirium.* Psychosomatics, 2016. **57**(3): p. 246-55.

9. Warren, N., et al., *Electroconvulsive therapy for anti-N-methyl-d-aspartate (NMDA) receptor encephalitis: A systematic review of cases.* Brain Stimul, 2019. **12**(2): p. 329-334.

10. Austgen, G., et al., *The Use of Electroconvulsive Therapy in Neuropsychiatric Complications of Coronavirus Disease 2019: A Systematic Literature Review and Case Report.* J Acad Consult Liaison Psychiatry, 2022. **63**(1): p. 86-93.

11. DeJong, H., P. Bunton, and D.J. Hare, *A systematic review of interventions used to treat catatonic symptoms in people with autistic spectrum disorders.* J Autism Dev Disord, 2014. **44**(9): p. 2127-36.

12. Vaquerizo-Serrano, J., et al., *Catatonia in autism spectrum disorders: A systematic review and meta-analysis.* Eur Psychiatry, 2021. **65**(1): p. e4.

13. Døssing, E. and A.K. Pagsberg, *Electroconvulsive Therapy in Children and Adolescents: A Systematic Review of Current Literature and Guidelines.* J ect, 2021. **37**(3): p. 158-170.

14. Jaimes-Albornoz, W., et al., *Catatonia in older adults: A systematic review.* World J Psychiatry, 2022. **12**(2): p. 348-367.
